# Volumetric modulated arc therapy for total body irradiation: A feasibility study using Pinnacle^3^ treatment planning system and Elekta Agility™ linac

**DOI:** 10.1002/acm2.12257

**Published:** 2018-01-24

**Authors:** Kirsty Symons, Colm Morrison, Jason Parry, Simon Woodings, Yvonne Zissiadis

**Affiliations:** ^1^ Genesis Cancer Care WA Department of Radiation Oncology Fiona Stanley Hospital Murdoch WA Australia; ^2^ School of Physics The University of Western Australia Perth WA Australia; ^3^ Department of Radiotherapy University Medical Center Utrecht Utrecht The Netherlands; ^4^ School of Surgery The University of Western Australia Perth WA Australia

**Keywords:** total body irradiation (TBI), total marrow irradiation (TMI), volumetric arc therapy (VMAT)

## Abstract

A study was undertaken to explore the use of volumetric modulated arc therapy (VMAT) for total body irradiation (TBI). Five patient plans were created in Pinnacle^3^ using nine 6 MV photon dynamic arcs. A dose of 12 Gy in six fractions was prescribed. The planning target volume (PTV) was split into four subsections for the head, chest, abdomen, and pelvis. The head and chest beams were optimized together, followed by the abdomen and pelvis beams. The last stage of the planning process involved turning all beams on and performing a final optimization to achieve a clinically acceptable plan. Beam isocenters were shifted by 3 or 5 mm in the left–right, anterior–posterior, and superior–inferior directions to simulate the effect of setup errors on the dose distribution. Treatment plan verification consisted of ArcCheck measurements compared to calculated doses using a global 3%/3 mm gamma analysis. All five patient plans achieved the planning aim of delivering 12 Gy to at least 90% of the target. The mean dose in the PTV was 12.7 Gy. Mean lung dose was restricted to 8 Gy, and a dose reduction of up to 40% for organs such as the liver and kidneys proved feasible. The VMAT technique was found to be sensitive to patient setup errors particularly in the superior–inferior direction. The dose predicted by the planning system agreed with measured doses and had an average pass rate of 99.2% for all arcs. VMAT was found to be a viable treatment technique for total body irradiation.

## 1 INTRODUCTION

Total body irradiation (TBI) is used in the management of hematologic malignancies prior to the transplantation of hematopoietic or bone marrow stem cells. The combination of radiation and chemotherapy kills the malignant cells, increasing the likelihood of a successful transplant and suppresses the recipient's immune system to prevent immunologic rejection. The prescription dose can range from 12 to 15 Gy in 6–12 fractions over 4–6 days in myeloablative approaches, with the most common prescription being 12 Gy in six fractions.^1^, ^2^, ^3^ Low‐dose TBI (2–8 Gy in 1–4 fractions) can also be used as an effective form of conditioning for older patients who may not be able to tolerate myeloablation.^1^, ^4^, ^5^ The most significant organ toxicity associated with TBI is lung toxicity. The lungs are typically shielded to <10 Gy to reduce the chance of radiation induced interstitial pneumonitis.^6^ Other key organs at risk from TBI include the liver, kidneys, spleen, heart, and eyes.^7^

TBI is traditionally treated using a conventional linac (linear accelerator) using static anterior–posterior/posterior–anterior (AP/PA) or parallel‐opposed lateral beam arrangements at extended source‐to‐surface distance (SSD). Photon beam energies between 4 and 24 MV can be used with tissue compensators to boost regions of varying patient thickness or shielding blocks to limit dose to organs at risk (e.g., lungs, liver, and kidneys). The dose is prescribed to a single point at the midline of the patient with the aim of delivering a uniform dose of ±10%.^8^ There are certain drawbacks associated with conventional techniques that have been well reported in the literature;^8^, ^9^ long treatment and setup times (which impact patient comfort and ability to maintain accurate positioning during treatment); a lack of accurate three‐dimensional treatment planning data; and the requirement for large linac bunkers to accommodate extended SSDs; and specialized treatment equipment.

Other TBI treatment methods include positioning the patient supine and prone on a fixed couch at a large SSD underneath the linac and delivering modulated partial arcs.^10^ This method offers greater dose uniformity; however, it is both time and labor‐intensive for planning and treatment delivery. A translational couch technique maintains a static beam and gantry, while the patient travels under the linac on a specialized translational couch.^11^ A homogeneous dose can be delivered by varying the speed of the couch movement.^12^, ^13^ Custom‐made lung shields and beam spoilers may still be required for these techniques.

Recently, there has been a shift toward more advanced TBI treatments utilizing modulated arc techniques to target the hematopoietic tissues and reduce the dose to the surrounding healthy tissues. Helical tomotherapy (HT) can be used to treat TBI patients.^14^, ^15^, ^16^ It has the advantage of the patient positioned supine, a homogeneous dose distribution without the need for junctions between beams, and with the ability to spare organs at risk. Total marrow irradiation (TMI) utilizes HT^14^, ^17^, ^18^ or volumetric modulated arc therapy (VMAT) on conventional linacs^19^, ^20^, ^21^ to treat the bone marrow itself, while reducing dose to the surrounding organs at risk and healthy tissue. Total marrow plus lymphoid irradiation (TMLI) targets the total marrow volume plus major lymph node chains, liver, spleen, and sanctuary sites such as the brain. In contrast to traditional TBI, this TMI technique has the potential to reduce both acute and chronic toxicities, reduce treatment time, increase patient comfort, and reduce the need for specialized equipment such as beam spoilers, shielding, and treatment frames. Dose escalation to the total marrow while limiting doses to normal organs to levels lower than in conventional TBI is currently being investigated. Wong et al.^22^ reported dose escalation to 15 Gy combined with cyclophosphamide and etoposide therapy is associated with acceptable toxicities and encouraging outcomes in patients with advanced acute leukemia undergoing bone marrow transplantation. Further clinical trials are required to determine appropriate TMI and TMLI doses and whether dose escalation translates into improved control rates and survival.

The aim of this study was to investigate the feasibility of achieving clinically acceptable TBI plans with the Pinnacle^3^ treatment planning system (TPS) and accurate delivery using an Elekta Agility™ linac. Previous studies have demonstrated the use of Varian RapidArc in combination with the Eclipse TPS^23^ with promising results in the first clinical cases.^24^ A VMAT approach to TBI treatments has the potential to make TBI accessible to more clinical departments where equipment limitations or bunker size has restricted implementation. This work may also facilitate a move toward a TMI type treatment in future where the skeletal and hematopoietic tissues can be targeted and potentially receive an escalated dose regime as more clinical data become available.

## 2 METHODS AND MATERIALS

A retrospective analysis was performed on five patients. Computed tomography (CT) images were obtained in the head‐first supine (HFS) orientation from the top of the skull to mid‐thigh, with a 5 mm slice thickness (GE Healthcare, Optima™ CT580). The patients were simulated with a thermoplastic mask over the head and neck region and a full body vacuum bag for immobilization. A custom head rest that also supports the shoulders was used to help keep a neutral spine position and raise the chin from the chest. The patients have their arms at their sides, as tight as possible to facilitate reproducibility of positioning on treatment, and simplify the target for treatment planning. A second CT dataset in the feet‐first supine (FFS) orientation is required to accommodate irradiation of the lower limbs due to the maximum scan length of the CT (140 cm) and longitudinal table movement limits on treatment. Although not included in this planning study, it is intended for the legs to be treated with conventional AP/PA fields.

The planning target volume (PTV) was defined as the entire body, contracted to 5 mm below the skin. The patient datasets included in the planning study encompassed a range of patient shapes and sizes. Total PTV volume ranged from 39722 to 61900 cc and lung volume ranged from 2275 to 3534 cc. The planning aims were to deliver a uniform dose of 12 Gy to the PTV while limiting the mean lung dose to less than 8 Gy, and the mean kidney and liver doses to below 9 Gy. The PTV was extended 3 mm into the lungs in a pragmatic compromise between coverage of setup, geometric, and intrafraction motion uncertainties and sparing of the lungs. The 3 mm margin was chosen based on reported margins used in TMI.^14^, ^18^, ^20^, ^21^, ^25^ A planning volume at risk (PRV) margin of 7 mm was applied to the kidneys in the superior–inferior direction to account for organ motion during treatment.^26^

**Figure 1:**
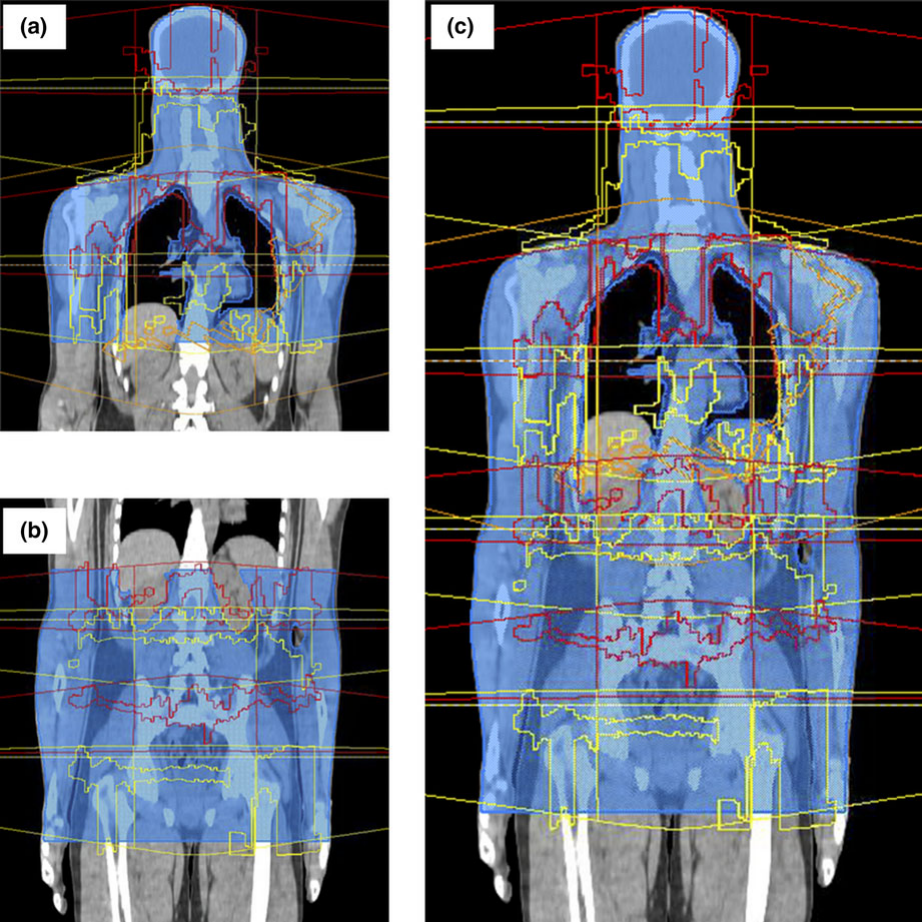
The beam arrangement and stages for TBI planning in Pinnacle. Nine VMAT arcs are arranged longitudinally along the patient and assigned to four isocenters. Each isocenter has a superior (red) and inferiorly (yellow) offset arc. The chest isocenter has an extra arc with no field size restrictions (orange). (a) Step 1: of the planning process is to optimize and convert the head and chest beams together. (b) Step 2: head and chest beams are set to “None” in IMRT parameters and the abdomen and pelvis beams are optimized together. (c) Step 3: All beams are turned on and a final optimization process performed to smooth out junction regions.

The Pinnacle^3^ SmartEnterprise version 9.10 (Philips Healthcare, Andover, MA, USA) treatment planning system was used to optimize VMAT beams. The system consists of three application servers with dual 2.93 GHz Intel Xeon 5600 series processor and 96 GB of RAM. The PTV was split into subsections for the head, chest, abdomen, and pelvis. A combination of nine 6 MV photon beams were arranged along the patient's longitudinal axis using an isocenter at the middle of each sub‐PTV. All isocenters had the same lateral and anterior–posterior coordinates to limit the couch moves required on treatment to only longitudinal shifts. The head, abdomen, and pelvis subsections each had two VMAT arcs rotating through 356^o^. The two VMAT arcs were offset in the superior and inferior direction around the isocenter. The maximum width of the field was set to 40 cm and the field length restricted to a maximum of 18 cm with a 4 cm overlap region at the isocenter. The collimator was rotated to 90^o^ for each of these offset arcs. An additional arc was required to achieve the planning aims in the chest PTV. The 356^o^ arc had a collimator rotation of 30^o^, with no restrictions on jaw movement. Figure 1 shows the offset beam arrangement for VMAT arcs.

Due to limitations of our Pinnacle^3^ system at optimizing and converting more than five beams concurrently over a large volume, the planning process for the nine VMAT arcs was broken into three stages. First, the head and chest beams were optimized and converted together. A maximum of 60 iterations were set with the optimizer given an initial “warm start” of 29 iterations before setting the dose convolution at 30 iterations. The head and chest beams were then switched off and the process repeated to optimize and convert the abdomen and pelvis beams. In the final stage of the process, all beams are switched on and optimized to smooth out junctions and any hot or cold spots. Typically, approximately 15 iterations are required to gauge the impact of a change in objective or constraints. The planning objectives and constraints set are shown in Table 1. A uniform dose objective with a high weighting was set to keep dose uniformity in the PTV to acceptable levels. Max EUD (equivalent uniform dose) objectives were set for the organs at risk. To maintain acceptable dose coverage to the ribs, the liver and lung objective ROIs were contracted by 5 mm (Liver_cont and Lungs_cont).

**Table 1:** Pinnacle^3^ optimization objectives used for VMAT TBI

ROI	Optimization type	Target (cGy)	% Volume	Weight	a
ptv_VMAT	Min DVH	1200	98	98	–
ptv_VMAT	Max DVH	1320	5	50	–
ptv_VMAT	Uniform dose	1220	–	85	–
Liver_cont	Max EUD	750	–	20	2
Kidneys_prv	Max EUD	750	–	20	2
Lungs_cont	Max EUD	680	–	40	3
ptv_skeleton	Min DVH	1200	98	100	–

During the optimization process, the air cavities within the patient were overridden to a density of 1 g/cm^3^, to restrict the optimizer from over increasing the photon fluence in low‐density regions. The air cavity density override was switched off for the final dose computation. Initial optimization and conversion was performed with a dose grid of 5 × 5 × 5 mm. The final dose computation was performed with a dose grid of 3 × 3 × 3 mm using the collapsed cone convolution algorithm.

Treatment plan evaluation was performed using dose–volume histogram (DVH) analysis. The planning aims for the PTV were to deliver 12 Gy to at least 90% of the PTV (i.e., V_100%_ ≥ 90%) and 11.4 Gy to at least 95% of the PTV (i.e., V_95%_ ≥ 95%). For the PTV, the mean dose was recorded as well as the hottest dose to 2 (D_2 cc_) and 5 cc (D_5 cc_) of the volume as an indicator of maximum dose. Organ at risk dose was assessed based on the mean dose to the organ. All reported doses to organs at risk are based on the outlined organ on the planning CT with no margins applied. The dose homogeneity of the target was reported as the ratio of the dose received by 90% of the volume (D_90%_) to the minimum dose received by the “hottest” 10% of the volume (D_10%_).^14^, ^27^

For a single patient plan, a series of shifts of 3 and 5 mm were applied to the isocenter position of the beams in the TPS to simulate potential setup errors on treatment. The dose was recalculated keeping the same monitor units as the original plan. The shifted plans were analyzed using DVH metrics and normalized to the reference plan. To analyze PTV coverage, the change to the mean dose, V_100%_, V_95%_, and the maximum dose was recorded. Mean dose values were used to assess the effect on OARs. For shifts applied to the chest beams, DVH data for the skeleton plus a 3 mm margin in the chest PTV were also recorded to evaluate the effect of setup errors on the rib coverage with the 3 mm margin. A shift in two directions (left–right and anterior–posterior) was tested using 5 mm for the head and pelvis beams and 3 mm for the chest and abdomen. The scenario of a 3 mm shift in all directions was also tested for all beams.

**Figure 2:**
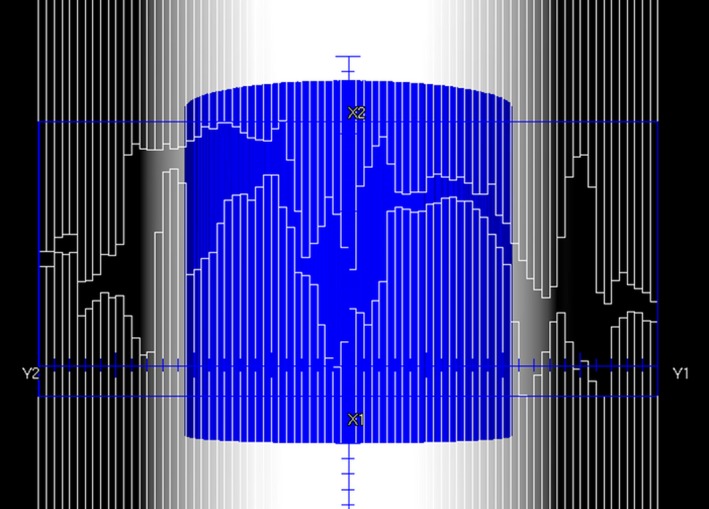
A “beams eye view” of a chest arc on the ArcCheck phantom. Both a left and right 8 cm shift was applied to the ArcCheck during measurement to capture the open segments at the field edges. The blue ROI represents the internal diode volume.

All VMAT arcs were measured on the ArcCheck (Sun Nuclear Inc, Melbourne, FL, USA) cylindrical diode array. The treatment plans were copied to the ArcCheck phantom in Pinnacle^3^ and calculated on a 2 × 2 × 2 mm dose grid. During measurement, the ArcCheck was shifted by ±6 cm longitudinally for the superior and inferior offset beams to allow all segments to be captured on the ArcCheck and to avoid irradiating the electronics. Due to the 90^o^ collimator rotation and the width of the fields, not all open beam segments are able to be captured on the ArcCheck when the device is positioned centrally (Fig. 2). To verify all segments and test the beam model and MLC calibration at the field edges, the ArcCheck was shifted by 8 cm laterally in both directions and compared to the same shift made on the phantom calculation in the planning system for the superior and inferior chest arcs only. All measurements were analyzed in the SNC Patient software (version 6.6.2) using a global gamma analysis of 3%/3 mm on absolute dose.

## 3 RESULTS

**Figure 3:**
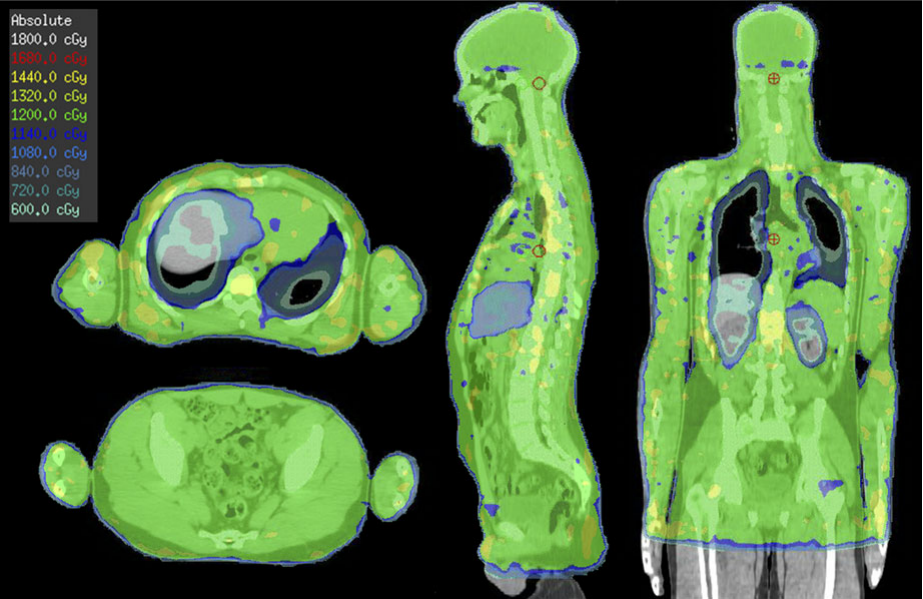
Isodose distribution of patient 3 for the chest and pelvis in the axial view, and a sagittal and coronal view.

An example dose distribution for patient 3 is shown in Fig. 3. The planning results achieved are given in Tables 2, 3, 4. The planning aims for the PTV were to deliver 12 Gy to at least 90% of the PTV (i.e., V_100%_ ≥ 90%) and 11.4 Gy to at least 95% of the PTV (i.e., V_95%_ ≥ 95%). This was achieved for each of the test plans. The mean dose to the lungs was able to be restricted to less than 8 Gy for each test case and over a range of total lung volumes. A 30% dose reduction to the liver and a 40% dose reduction to the kidneys proved to be feasible, while the maximum dose and dose homogeneity were kept to acceptable ranges.

**Table 2:** Planning outcomes for the PTV

Patient	PTV Volume (cc)	Mean (Gy)	V_100%_	V_95%_	D_2 cc_ (Gy)	D_5 cc_ (Gy)	D_90%_ (Gy)	D_10%_ (Gy)	Homogeneity (D90/D10)
1	39722	12.6	95.5	98.3	14.9	14.7	12.2	13.2	0.93
2	41082	12.8	96.1	98.2	14.5	14.4	12.3	13.2	0.93
3	44847	12.6	93.4	97.6	15.0	14.8	12.2	13.2	0.92
4	45458	12.6	92.1	97.2	14.9	14.7	12.1	13.1	0.92
5	61900	12.7	94.4	98.2	15.4	15.6	12.2	13.5	0.90

**Table 3:** Percentage volume in the PTV receiving 90%, 100%, 105%, 110%, and 120% of the prescription dose

Patient	Volume (%)				
	90%	100%	105%	110%	120%
1	99.1	95.5	50.5	6.6	0.03
2	99.0	96.1	71.6	10.5	0.01
3	98.7	93.4	57.9	8.6	0.08
4	98.3	92.1	48.7	7.5	0.06
5	98.7	94.4	70.2	20.4	0.46

**Table 4:** Planning outcomes for the organs at risk

Patient	Lungs		Liver	Kidneys	
	Lung Volume (cc)	Mean (Gy)	Mean (Gy)	Lt Mean (Gy)	Rt Mean (Gy)
1	2275	7.8	8.6	6.0	6.9
2	3396	7.7	8.1	7.4	6.9
3	2671	7.8	8.0	7.7	6.7
4	2236	7.4	7.5	6.2	6.2
5	3534	7.1	7.6	7.3	7.4

Tables 5 and 6 present the results for the shifted plan data. Results were grouped and averaged depending on whether OARs were present in the PTV. No significant deviations for PTV coverage were found when shifts of up to 5 mm were applied in the left–right (L‐R) or anterior–posterior (A‐P) direction. PTV coverage, specifically V_100%_, was found to be impacted most by shifts in the superior–inferior (S‐I) direction. A 5 mm shift in the S‐I direction can have on average an 11% change to V_100%_ in the head and pelvis PTVs or 8% in the chest and abdomen PTV. Similarly, OAR mean dose is most sensitive to S‐I shifts, with a 5 mm shift causing a change of 5% from the planned mean dose. Rib coverage was maintained in the chest PTV for L‐R and A‐P shifts of 5 mm but V_100%_ decreased significantly for S‐I shifts. No significant deviations to PTV coverage were found when the beams are shifted in two directions and the S‐I isocenter value is kept constant. The plans were observed to be hotter at the edges of the PTV with the maximum dose increased by 3% on average. Mean kidney dose increased by up to 3.6%. For the scenario of a 3 mm shift in all directions, the results are comparable to the 3 mm S‐I only shift data, with the largest effect on V_100%_ of the ribs. Results from the verification of the patient treatment plans are given in Table 7. The average gamma pass rate for all arcs (nine beams per patient) measured with a 0 cm lateral offset is 99.2%. For individual arcs, the pass rate ranged from 95.7% to 100%. The average pass rate for the 8 cm lateral offset chest arcs was 98.7% and ranged from 96.9% to 99.7%.

**Table 5:** DVH analysis for the head and pelvis PTVs relative to the original treatment plan

	Head & Pelvis						5, 5, 0 mm	3, 3, 3 mm
	L‐R 3 mm	L‐R 5 mm	A‐P 3 mm	A‐P 5 mm	S‐I 3 mm	S‐I 5 mm		
PTV								
Mean	100.0	99.9	100.0	99.8	99.9	99.1	100.0	99.8
V_100%_	99.8	99.1	99.8	98.9	95.3	88.5	99.1	95.1
V_95%_	100.0	99.8	100.0	99.9	99.8	97.5	99.9	99.7
Max dose	100.9	98.8	99.2	101.4	100.0	99.8	103.1	101.7

**Table 6:** DVH analysis for the chest and abdomen PTVs relative to the original treatment plan

	Chest & Abdomen						3, 3, 0 mm	3, 3, 3 mm
	L‐R 3 mm	L‐R 5 mm	A‐P 3 mm	A‐P 5 mm	S‐I 3 mm	S‐I 5 mm		
PTV								
Mean	99.9	99.8	100.0	99.9	100.0	100.1	100.2	99.5
V_100%_	99.6	99.0	99.9	99.4	96.2	92.3	100.4	94.0
V_95%_	99.8	99.3	100.0	99.7	100.2	99.1	100.1	98.4
Max dose	99.9	100.0	99.5	99.7	100.8	102.2	100.1	100.5
OAR (mean dose)								
Lungs	100.3	100.7	100.2	100.6	101.7	104.0	101.3	99.9
Liver	100.2	100.5	99.8	99.7	103.1	105.3	99.7	97.5
Kidney L	100.5	102.2	100.5	101.8	102.4	104.9	102.4	99.1
Kidney R	98.3	98.4	100.2	101.3	101.9	103.8	103.6	97.6
Skeleton + 3 mm								
Mean	99.8	99.4	99.9	99.8	99.9	98.9	100.5	99.6
V_100%_	99.7	98.5	101.6	101.5	91.8	86.2	101.5	93.7
V_95%_	99.4	98.1	100.7	100.4	99.6	91.7	100.5	96.5

**Table 7:** ArcCheck Measurement results for the five patient test plans

Patient	0 cm lateral offset – All Beams *γ* (3%/3 mm)		±8 cm lateral offset – Chest beams *γ* (3%/3 mm)	
	Average	Std Dev	Average	Std Dev
1	99.5	0.5	99.2	0.8
2	99.7	0.5	98.4	1.1
3	99.0	0.9	99.2	0.3
4	99.0	1.4	99.0	0.8
5	98.6	0.7	97.9	1.1

## 4 DISCUSSION

The use of rotational intensity modulation is becoming increasing popular for all radiotherapy treatment sites due to the rapid treatment delivery times and the ability to conform dose to complex targets while sparing critical structures. Rotational techniques using conventional linacs are yet to be fully utilized for TBI due to the complexities involved in treating an extremely large target with multiple overlapping arcs. In this study, we present a technique for planning and treating TBI patients using the combination of Pinnacle^3^ TPS and the Elekta Agility^™^ linac.

The planning results achieved demonstrate a VMAT approach to TBI is able to deliver a dose prescription of 12 Gy to the PTV, while sparing the mean lung dose to 8 Gy or less. VMAT also offers the ability to selectively spare other organs at risk such as the kidneys, liver, and brain, or even account for areas of previous treatment. Although the dose to the liver and kidneys has been reduced for all patients in this study, organ sparing should be evaluated on a case by case basis. The liver for example, is often included in the target of TMLI studies as it may contain hematopoietic tissues. The hottest dose to 2 cc of the volume was recorded and dose homogeneity index was calculated for the VMAT plans using D_90%_/D_10%_. D_2 cc_ doses of up to 130% and the dose homogeneity index within 10% were in keeping with results reported by other modulated VMAT techniques.^14^, ^24^ Although no strict limit is set on D_2 cc_ or the dose homogeneity, regions of hotspots are evaluated based on their location in the patient. Hotspots may be acceptable if they are away from critical structures and mostly in external tissues. Due to the normalization of the PTV dose (V_100%_ ≥ 90%), the average dose is shifted higher than the prescribed dose for the VMAT plans (mean dose in the PTV over the five patients was 12.7 Gy). In this study, the PTV was trimmed to 5 mm below the surface of the patient as certain problems arise when attempting achieve the planned absorbed dose in this region. Within the buildup region and with the coarse voxel resolution used during optimization (5 × 5 × 5 mm), the dose increases rapidly and has large uncertainty. It is difficult to achieve the planned dose in this region due to the lack of electronic equilibrium; the inverse optimizer must therefore increase the dose in this region by strongly increasing the photon fluence which can lead to reduced homogeneity in the PTV. Hot spots can also be created by small setup errors during treatment. For the above reasons, it is also difficult to assess the skin dose, but in practice the combination of multiple arcs, oblique beam incidence, and beam exit from all angles significantly reduces the normal photon beam skin‐sparing effect. Although not included in this study, skin dose could be boosted by using well‐documented techniques such as bolus, a beam spoiler, or a virtual bolus in the planning system.^28^

The practice of delivering TBI at low dose rates stems from radiobiological considerations, namely the sparing of damage as a result of cellular recovery.^29^, ^30^ Pneumonitis is one of the major toxicity concerns with TBI, and was originally related to dose rate; however, since the introduction of fractionated regimes, several publications have shown the dose rate to have little effect.^31^, ^32^, ^33^, ^34^, ^35^, ^36^ The estimated dose rate in the chest region for this technique is 26 cGy/min, which is within the range of dose rates reported in the literature.^2^, ^3^ Although not restricted in this study, dose rate could potentially be limited by creating a TBI‐specific beam model with reduced maximum dose rate and gantry speed.

The data presented for simulated treatment shifts reiterate the importance of accurate patient positioning in TBI VMAT, particularly in the superior–inferior direction. Daily image‐guided radiotherapy with online couch adjustment should be used for all isocenters to minimize positional uncertainties. A less stringent tolerance in the L‐R and A‐P directions may be acceptable in regions where there are no organs at risk to spare, such as the head and pelvis in this technique. The location of junction regions in the S‐I direction should be chosen carefully to avoid potential overdosing of critical organs such as the lungs, or underdosing of regions of large bone marrow volume such as the femurs.

Initial measurements of TBI VMAT beams on the ArcCheck highlighted areas for improvement in our Agility™ beam model in Pinnacle^3^. Prior to the beam model update the majority of our pass rates for test TBI beams were greater than 95% but several beams, particularly the highly modulated chest arcs, had pass rates in the low 80%–85% range. This had not been observed in measurements of our clinical VMAT cases where pass rates are generally greater than 95%. The leaf offset table was investigated due to the beam setup and limitations placed on the TBI arcs. As arc pairs are offset from each other in the superior and inferior direction, and have a 90^o^ collimator rotation, there is a large amount of MLC leaf travel required over the beam central axis (Fig. 2). Improvements were made to the leaf offset table beyond ±10 cm to improve the planning system accuracy, with the table now more closely resembling the most recent Pinnacle^3^ recommendations for Agility™ machines.^37^ Pass rates for the newly commissioned beam model for TBI arcs were found to be on average 99.2%. As such, the tolerance for TBI VMAT QA will be equivalent to normal clinical VMAT cases (i.e., a pass rate greater than 95% is required for each arc). Due to the beam setup and field widths, not all segments can be captured in a single ArcCheck measurement. A lateral offset of ±8 cm was applied to the superior and inferior chest arcs to verify the beam model and MLC calibration at the field edges. The results exhibited clinically acceptable pass rates and were comparable to measurements with the ArcCheck centered laterally. For ongoing routine pretreatment QA, it is expected a measurement with the ArcCheck centered will be sufficient provided a thorough MLC QA program is in place.

It is important to note the irradiation of the lower limbs was not included in this paper. It is intended the legs be treated in the feet‐first direction using AP/PA beams with conventional static fields. The ideal method would be to treat the lower legs with a series of VMAT arcs also to smooth doses in the junction regions. This has proved a challenge due to the difficulties in junctioning two VMAT arcs that have been planned on two CTs with different treatment orientations (i.e., head‐first and feet‐first orientation). Although Springer et al.^24^ have described a method for irradiating the legs with VMAT by summating the resulting head‐first and feet‐first plans in Eclipse and performing a final optimization of the junction region. Junctioned AP/PA beams for the legs are deemed acceptable owing to the absence of any organs at risk, and are the simplest approach to achieving the prescribed dose to the target.

Moving to a VMAT TBI technique will be both labor and resource intensive. CT simulation is estimated to take between 1 and 1.5 hr. The contouring of required PTVs and organs at risk is expected to take approximately 2 hr using the auto‐contouring software MIM Maestro™ (MIM software). Total time for optimization of the nine VMAT arc is approximately 21 hr (or 3–4 working days), but requires minimal user input with automated scripting in Pinnacle^3^. This does not include the time required for planning of the legs, plan checking, and export to the record and verify system. However, total time on the treatment machine will be decreased as appointments for simulation and imaging checks on the shielding blocks will no longer be required. It is estimated the time per treatment fraction will be reduced also from 1.5 to 2 hr for the 2D extended SSD technique to 1–1.5 hr per fraction for VMAT. The total time for physics quality assurance is 6–8 hr for dose calculation and 2–3 hr of machine measurements.

## 5 CONCLUSION

This study has demonstrated the feasibility of achieving clinically acceptable VMAT TBI plans with the Pinnacle^3^ treatment planning system and accurate delivery using an Elekta Agility™ linac. The advantages of this technique include improved patient comfort and positioning reproducibility during treatment, accurate 3D dose information, and the ability to selectively spare organs at risk.

## CONFLICT OF INTEREST

The authors declare no conflict of interest.
